# In vitro evidence of efficacy and safety of a polymerized cat dander extract for allergen immunotherapy

**DOI:** 10.1186/s12865-017-0193-0

**Published:** 2017-02-24

**Authors:** María Morales, Mayte Gallego, Victor Iraola, Marta Taulés, Eliandre de Oliveira, Raquel Moya, Jerónimo Carnés

**Affiliations:** 1 Research & Development, Laboratorios LETI, S.L., Calle del Sol n° 5, 28760 Madrid, Tres Cantos Spain; 2 Centres Científics i Tecnològics, Universitat de Barcelona, Barcelona, Spain; 3 Plataforma de Proteòmica, Parc Científic de Barcelona, Barcelona, Spain

**Keywords:** Cat, Immunotherapy, Surface plasmon resonance, Cell studies, Dander

## Abstract

**Background:**

Allergy to cat epithelia is highly prevalent, being the major recommendation for allergy sufferers its avoidance. However, this is not always feasible. Allergen specific immunotherapy is therefore recommended for these patients. The use of polymerized allergen extracts, allergoids, would allow to achieve the high allergen doses suggested to be effective while maintaining safety.

**Results:**

Cat native extract and its depigmented allergoid were manufactured and biochemically and immunochemically characterized. Protein and chromatographic profiles showed significant modification of the depigmented allergoid with respect to its corresponding native extract. However, the presence of different allergens (Fel d 1, Fel d 2, Fel d 3, Fel d 4 and Fel d 7) was confirmed in the allergoid. Differences in IgE-binding capacity were observed as loss of biological potency and lower stability of the IgE-allergen complex on surface plasmon resonance. The allergoid induced production of IgG antibodies able to block IgE-binding to native extract. Finally, studies carried out with peripheral-blood mononuclear cells from cat allergic patients showed that the allergoid induced IFN-γ and IL-10 production similar to that induced by native extract.

**Conclusions:**

Cat depigmented allergoid induced production of cytokines involved in a Th1 and Treg response, was able to induce production of IgG-antibodies that blocks IgE-binding to cat native extract, and showed reduced interaction with IgE, suggesting greater safety than native extract while maintaining in vitro efficacy.

## Background

Sensitization to cat dander presents a high prevalence, affecting more than 25% of the population in Western countries [1] and more than 15% in the US [2]. Around half of sensitized patients may present symptoms [3], being rhinoconjunctivitis the most frequent symptom.

Apart from avoidance strategies or symptomatic treatment, cat allergy is currently treated with immunotherapy consisting of the administration of native extracts (NE) either via subcutaneous or sublingual routes of administration. The use of cat native extracts has demonstrated clinical efficacy and safety profile in several clinical trials [4, 5], being the efficacy related to Fel d 1 content [6], and the amount of extract used is in turn related to adverse reactions [7]. The development of chemically modified allergen extracts by polymerization with glutaraldehyde has been postulated as an alternative in immunotherapy, maintaining/increasing the efficacy while reducing the risk of adverse reactions [8]. The resulting products are high molecular weight allergen chains which contain all the allergens present in NE in a polymerized form. An intermediate patented process known as “depigmentation” [9] consist on the purification of the NE for the removal of irrelevant allergenic substances with the objective to increase the concentration of the individual allergens. To date, this method has yielded good results in other aeroallergens, including mites and pollens [10, 11]. Other alternatives based on immunologically active peptides are currently under investigation [12, 13]. In addition, monomeric carbamylated allergoid sublingual immunotherapy also present good results [14].

In recent years, different techniques have been implemented in characterization of active substances used in immunotherapy, providing a clear advantage not only in the quality of immunotherapy treatments already in the market but also supporting and providing useful information for immunotherapeutic design and for the early stages of the development process. Measurement of IgE interaction with allergens by surface plasmon resonance (SPR) analysis has been postulated as an indicator of safety [15], while cellular assays based on cytokine release or quantification of IgE-blocking IgG-antibodies indicate immunological response suggesting efficacy. The design of immunotherapy based on immunological principles seems logical to guarantee the success of new products.

Based on these concepts, the objective of this study was to design and produce a depigmented allergoid of cat epithelium to be used in immunotherapy based on safety and efficacy data.

## Methods

### Allergen extract preparation

Two hundred grams of cat dander (Allergon, Sweden) were extracted in Phosphate Buffered Saline 0.01 M under continuous magnetic stirring, for 4 h at 4 °C. The resulting product was called native extract (NE). The NE was depigmented after a mild acid treatment (pH 2) followed by dialysis against bi-distilled water with a cut-off membrane of 3.5 kDa (Cellu Sep Membrane, Seguin, TX, USA) with the objective to remove the low molecular weight components. Finally the pH was adjusted to seven. The resulting depigmented extract was polymerized with glutaraldehyde at a concentration of 5 mg/ml and extensively dialyzed against bi-distilled water in 100 kDa dialysis membranes (Millipore, Bedford, USA) to remove non-polymerized compounds. Finally, the polymerized product was freeze-dried, obtaining the cat depigmented-polymerized extract (CDA). All extracts were manufactured in strict compliance with GMP principles, following internal procedures (Laboratorios LETI, Spain, [9]).

### Serum samples and PBMC

A pool of four sera was used to evaluate IgE-binding to allergen extracts. In that sense, sera from cat-sensitized individuals were purchased from Plasmalab International (WA, USA), which operates in full compliance with U.S. Food and Drug Administration regulations. Specific IgE titer to cat dander in the pool of sera were 66.4 KU/l, to Fel d 1 55.3 KU/l, to Fel d 2 6.43KU/l, and to Fel d 4 9.60 KU/l, performed in ImmunoCAP system (Thermo Fisher Scientific, MA USA). The pool of sera was negative for bromelain (0.05 KU/l).

Polyclonal antibodies were used for IgG-binding assays to allergen extracts. Specific antibodies were induced in two New Zealand white rabbits after three immunizations with 200 μg of CDA adsorbed onto 3% aluminium hydroxide. All procedures were approved by the Biolab Institutional Review Board (Biolab, S.L., Colmenar Viejo, Spain), and followed local ethics rules for animal experimentation.

Peripheral-blood mononuclear cell (PBMC) culture supernatants (Sanguine BioSciences, CA, USA, compliant with FDA regulations) from three asthmatic cat-atopic donors not previously treated with immunotherapy were used to evaluate the capacity to stimulate cytokine production.

### Allergen extract characterization

#### Protein content

The protein content of NE and CDA extracts was measured using the Lowry–Biuret method (Sigma Diagnostics, St. Louis, USA) following the manufacturer’s instructions.

#### Protein profile

One hundred micrograms of NE and CDA extracts were loaded in SDS–PAGE gels with 2.67% C, 15% T acrylamide under reducing conditions and stained with Biosafe Coomassie (Bio-Rad Laboratories, Hercules, CA, USA).

#### Allergenic profile

Proteins from previously prepared SDS–PAGE gels (see above) were transferred to an Immobilon®-P membrane (Millipore, Bedford, Mass., USA). Thereafter, the membrane was incubated overnight with a pool of sera from cat-sensitized individuals. Afterwards, the membrane was washed and finally incubated with monoclonal α-human-IgE-PO (Ingenasa, Madrid, Spain). Finally the reaction was developed with Immun-Star™ Western Kit (BioRad).

Fel d 1, the major allergen from cat, was identified using a similar methodology but using as a primary antibody α-Fel d 1 monoclonal antibody-biotin (Indoor Biotech, VA, USA). After washing, the membrane was incubated with streptavidin-peroxidase and finally developed with Immun-Star™ Western Kit.

#### Major allergen quantification

Major allergen, Fel d 1, was quantified in NE extract using a specific commercial kit (EL-FD1) following manufacturer´s instructions (Indoor Biotech, VA, USA). Data were adjusted to a four-parameter logistic curve by the least-squares method. Determination of Fel d 1 in CDA was estimated based on NE determination and yield.

#### Polymerization profile

CDA molecular weight distribution was determined by SDS-PAGE (AnyKD TGX Precast Gels, BioRad Laboratories, CA, USA) using a high-molecular weight standard (Thermo Fisher Scientific Inc, MA USA), and by high-performance size exclusion chromatography (SEC) using a Bio SEC-3 Column (Agilent Technologies, CA, USA) in a 1200 series HPLC system (Agilent), at 1 ml/min 150 mM phosphate buffer, pH 7. Detection was performed at UV-280 nm.

#### Allergen identification

The presence of relevant allergens in NE and CDA was determined by mass spectrometry. Briefly, extracts were digested with trypsin and the peptide mixture was analyzed in a nanoAcquity liquid chromatography system (Waters Corporation, MA, USA) coupled to a LTQ-Orbitrap Velos (Thermo) mass spectrometer. Raw data were collected with ThermoXcalibur software (Thermo). A database search was performed through the Mascot search engine using Thermo Proteome Discover against the Uniprot database.

#### In vitro safety

##### IgE affinity by Surface Plasmon Resonance

SPR measurements were performed on a Biacore T100 system (Biacore, Uppsala, Sweden). Briefly, α-human-IgE was immobilized through amine coupling onto a C1 chip (GE Healthcare, NJ, USA) and the reference flow cell was treated using the same chemicals but in the absence of antibodies. The pool of sera from cat-sensitized individuals was diluted 1:2 and injected to capture IgE. Finally, NE and CDA (0.4 mg/ml) were injected to determine the stability of the IgE-NE or IgE-CDA complexes by measuring the dissociation rate (k_d_) and half-life (t½ = ln2/k_d_) of the complex.

##### IgE binding capacity by determination of biological potency

Biological potency of the extracts was calculated by ELISA competition assays, as previously described [16]. Briefly, each extract is compared with the In House Reference Preparation (IHRP), previously in vivo standardized. Nunc microplates (Thermo Scientific) were coated with anti-IgE (Ingenasa, Madrid, Spain). A pool of sera from cat sensitized patients was incubated in the plate. Dilutions of the sample and IHRP were incubated with the allergen labelled with peroxidase. The mixture was added to the coated plate and incubated. Afterwards, development solution (chromogen) was added, stopped with sulfuric acid and measured at OD 450 nm. Percentage of loss of potency was calculated as difference of biological potency between NE and CDA divided by NE potency.

#### In vitro efficacy

##### IgE Blocking antibodies

The capacity of CDA to induce allergen-specific polyclonal IgG antibodies able to block IgE binding sites to the allergen was evaluated by ELISA inhibition, as previously described [17]. Briefly, microplates were coated with NE (2 μg/well). After incubation with the generated polyclonal antibodies against CDA, plates were incubated with the pool of sera from cat-sensitized individuals. A secondary antibody anti-human-IgE-PO (Ingenasa, Madrid, Spain) was used for detection at 405 nm. Percent of inhibition was calculated by comparing IgE binding after incubation with preimmune or final bleeding polyclonal antibodies. Briefly, CDA capacity to induce sIgG with capacity to inhibit IgE binding of patients serum to a cat epithelia NE was evaluated. Percentage of inhibition was calculated as follows: percentage of IgE binding = 100 − (ODf/OD$P$) × 100. ODf and OD$P$ correspond to the optical densities after the preincubation of serum with the rabbit’s final sera and the corresponding preimmune sera, respectively.

##### Cytokine production

The capacity to stimulate cytokine production in PBMCs was evaluated using a quantitative ELISA-based Q-Plex™ test (Quansys Biosciences, UT, USA), performed in accordance with the manufacturer’s instructions. PBMCs (2×10^5^ cells per well) from cat sensitized patients were stimulated in triplicate with NE or CDA extract (100 μg/ml), and the production of IL-4, IFN-γ, IL-10 and IL-17 cytokines was measured in culture supernatants at 24 and 72 h. Phosphate buffered saline (PBS, 50 ng/ml) and concanavalin A (Con A, 5 μg/ml) were used as negative and positive controls, respectively.

## Results

### Protein and major allergen content

Protein content estimated by the Lowry-Biuret method was 216 μg prot/mg in NE and 254 μg prot/mg in CDA. NE contained approximately 25 μg of Fel d 1/mg. The estimated Fel d 1 content in CDA was 48 μg/mg.

### Protein and allergen profile

**Fig. 1:**
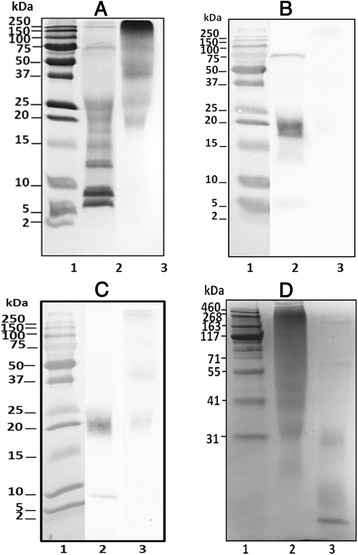
SDS-PAGE (**a**) and immunoblot (**b** and **c**) of cat epithelia in reducing conditions (15%T-2.67%C): Precision Plus Protein Dual Extra Standard (lane 1), NE (100 μg extract, lane 2) and CDA (100 μg, lane 3). Immunoblots were performed using serum from cat sensitized patients (**b**) or monoclonal antibody α-Fel d 1 (**c**) as primary antibody. High molecular weight SDS (**d**): HiMark^TM^ Pre-Stained HMW Protein Standard (lane 1), CDA (100 μg extract, lane 2), and NE (100 μg, lane 3)

The protein profile of NE (Fig. 1a) showed different bands of a wide range of molecular weight. The most prominent bands showed a low molecular weight (mainly 8 and 6 kDa). On the contrary, CDA showed higher molecular weight bands.

Allergenic profile was significantly different between NE and CDA (Fig. 1b), showing the most intense IgE-recognized band at 18 kDa in NE, coincident with Fel d 1 heterodimer (constituted by two subunits, of 4 and 14 kDa). Fel d 1 can also be found in a 36 kDa tetramer form. Fel d 1 band identity was confirmed by immunoblot using α-Fel d 1 monoclonal antibody (Fig. 1c). IgE binding to Fel d 1 was not observed in CDA (Fig. 1b), and α-Fel d 1 monoclonal antibody recognition was less intense (Fig. 1c).

### Polymerization profile

**Fig. 2:**
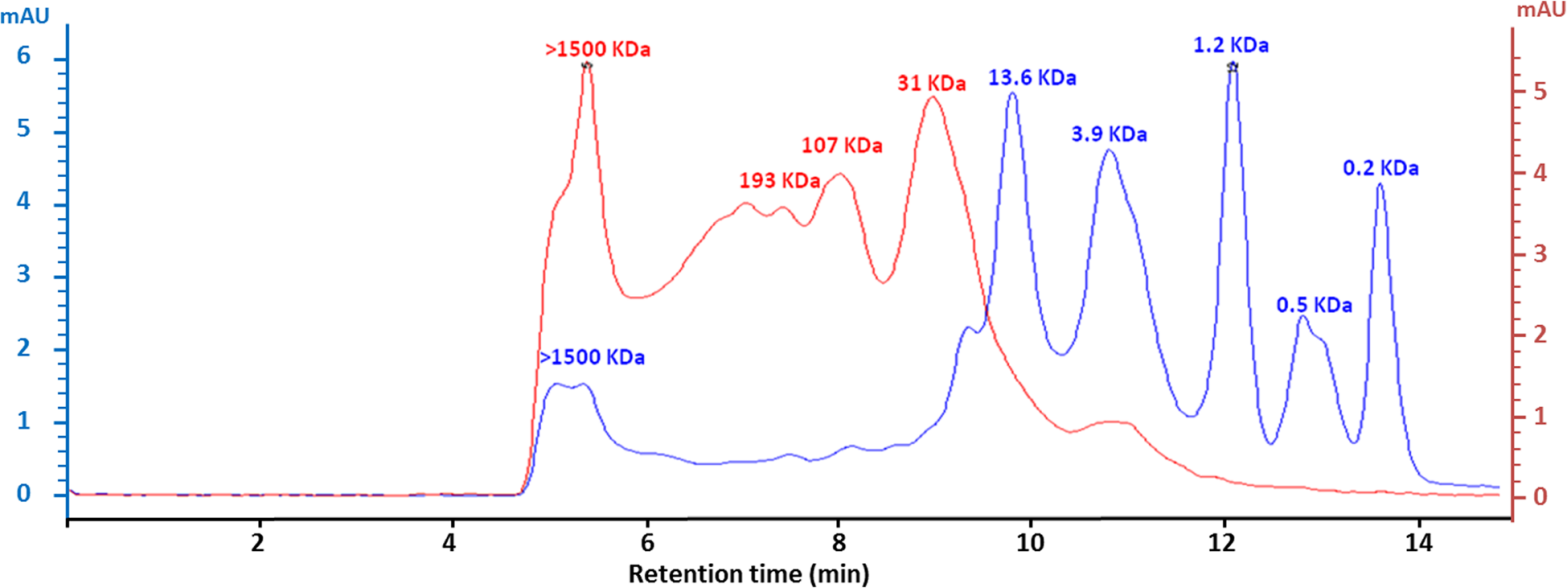
Size exclusion chromatogram: NE (*blue line*) and CDA (*red line*) detected at 280 nm (elution time in minutes). Optical density of NE is marked in *left* y-axe, and CDA in *right* y-axe

Specific methods (SDS-PAGE and SEC-HPLC) for detection of high molecular weight proteins were used to evaluate CDA polymerization profile (Figs. 1d and 2). Both methods showed a significant modification of CDA protein profile with respect to its corresponding NE. Low molecular weight proteins (at 4 and 14 kDa) were observed in NE but not in CDA (Fig. 1d. Proteins of approximately 31 and 107 kDa were observed in CDA chromatogram, although a high percentage of molecules exhibited a molecular weight higher than 1500 kDa (Fig. 2).

### Allergen identification

NE was sequenced by mass spectrometry, which confirmed the presence of Fel d 1, Fel d 2, Fel d 3, Fel d 4 and Fel d 7. The sequence coverage was 59% for chain 1 Fel d 1 (compared to Uniprot sequence code P30438), 40% for chain 2 Fel d 1 (Uniprot P30440), 51% for Fel d 2 (Uniprot M3WFW6), 69% for Fel d 3 (Uniprot Q8WNR9), for 37% Fel d 4 (Uniprot Q5VFH6) and 48% for Fel d 7 (Uniprot E5D2Z5). CDA sequencing showed the same allergens identified in NE.

CDA sequencing showed the same allergens identified in NE. Sequences coverage were 59% for chain 1 Fel d 1 (compared to Uniprot sequence code P30438), 26% for chain 2 Fel d 1 (Uniprot P30440), 45% for Fel d 2 (Uniprot P49064), 47% for Fel d 3 (Uniprot Q8WNR9), 29% for Fel d 4 (Uniprot Q5VFH6) and 52% for Fel d 7 (Uniprot E5D2Z5).

### In vitro safety

In vitro safety was evaluated as the reduced IgE binding capacity of CDA compared to NE. Two assays were planned. The first one was IgE binding to evaluate dissociation constant and half-life of the complex IgE-extract by Surface Plasmon Resonance. The second one was the loss of biological potency after polymerization. This was performed by ELISA competition assays comparing IgE binding capacity of NE and CDA to a IHRP.

The stability of the complex Antigen-IgE, measured by SPR, showed that the k_d_ of IgE-CDA was 8.0*10^−3^s^−1^ and the t_½_ was 87.22 s. In contrast, the k_d_ of IgE-NE was 1.9*10^−3^s^−1^, and the t_½_ was 360.2 s. The IgE-CDA complex was 4.1 times less stable than IgE-NE regarding the half life of the complex. In line with these results, polymerization induced a loss of biological potency higher than 95%. CDA presented a biological potency of 15 HEPL/mg, that represented a 99% of loss of biological potency compared to the NE (1035 HEPL/mg).

### In vitro efficacy

**Fig. 3:**
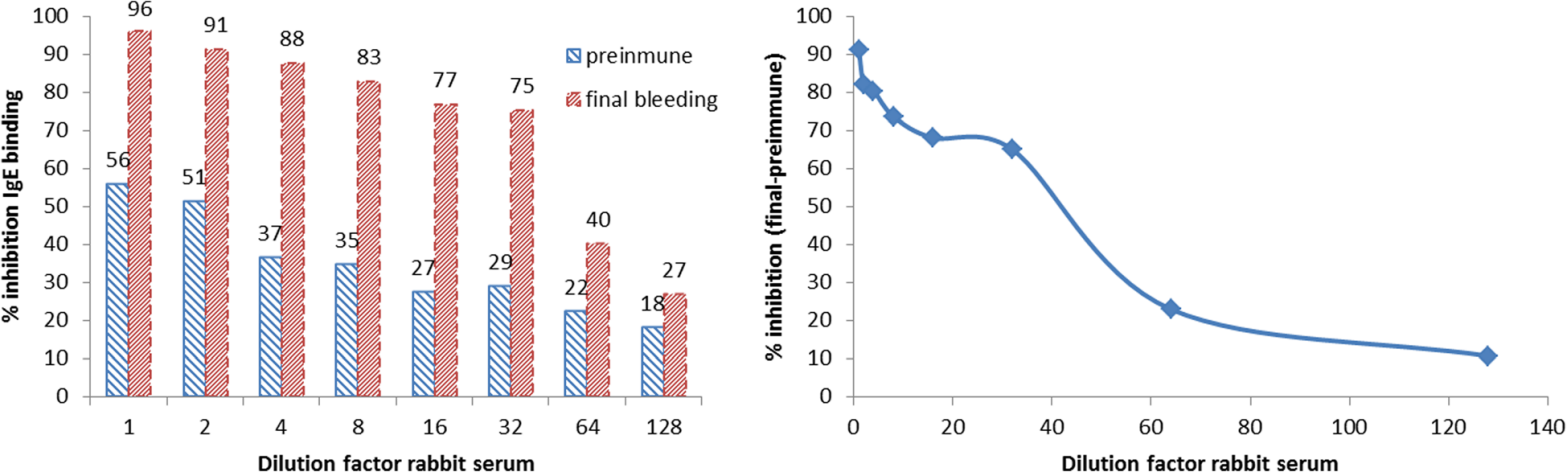
ELISA inhibition assay. Plates were coated with NE and incubated with polyclonal antibodies before human serum was added. Specific IgE binding to the plate was detected using anti-human-IgE-PO. Left: inhibition of preimmune and final bleeding serum samples. Right: inhibition of final bleeding compared to preimmune serum samples

CDA extract induced the production of IgG antibodies able to block up to IgE-binding sites. Specific IgE titers serums showed a low titer of sIgG titer (consequence of natural exposure to this allergen) lower than final bleeding samples. There was a 90% increase of induced inhibition comparing final and preimmune bleeding polyclonal samples (Fig. 3).

**Fig. 4:**
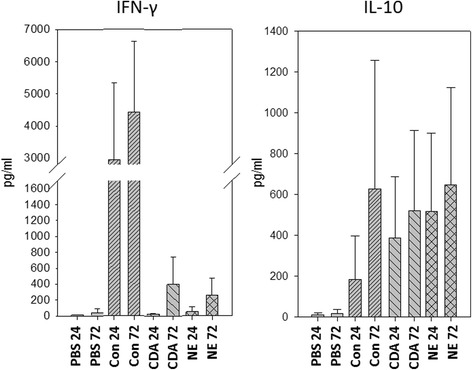
Mean value of the IFN-γ (left) and IL-10 (right) induction (in pg/ml) by PBMCs from cat-allergic donors (N = 3) after 24 or 72 h of treatment with PBS (negative control), concanavalin A (positive control), cat depigmented allergoid (CDA) or native cat extracts (NE). Error bars refer to standard deviation

Cellular studies showed a similar production of IFN-γ and IL-10 (Fig. 4) by NE and CDA extracts. After 72 h, NE induced levels of 258.1 pg IFN-γ/ml and 648.7 pg IL-10/ml, while CDA induced 394.7 pg IFN-γ/ml and 520.7 pg IL-10/ml. IL-17 and IL-4 were not detected in any case (values below the limit of quantification).

## Discussion

Cat allergy is one of the most prevalent allergic diseases in Western countries [1, 2]. Although limited clinical studies and numerous clinical observations have demonstrated the efficacy of cat immunotherapy with native extracts [4–6, 18], the adverse reactions associated to these allergenic extracts remains being an issue to be solved. To our knowledge, this is the first time that a polymerized allergenic extract is manufactured and its safety and efficacy deeply evaluated by in vitro techniques. The manufacturing process was designed based of the reduction of the IgE binding capacity, but also to increase the immunological and clinical efficacy of the product by confirming the presence of the relevant allergens and the stimulation of the appropriate immunological pathways. The present study confirmed that CDA was immunologically active and able to induce a Th1/Treg response. Moreover, it also induced the production of IgG blocking antibodies, inhibiting the IgE binding capacity of serum obtained from allergic patients. In addition, CDA was also less allergenic, as its binding capacity to IgE was lower than that of NE.

In recent years, many studies have tried to elucidate the mechanism of action of immunotherapy. It has been suggested that successful allergen immunotherapy should be accompanied by induction of regulatory T cells (Treg) and shift from a Th2 response toward a Th1 response [19]. Additionally, recent studies have suggested that specific biomarkers for measurement of the success of immunotherapy should be related to the capacity of allergen vaccines to specifically stimulate production of IL-10 and IFN-γ, which are involved in the Th1 and Treg responses, respectively, and a reduction of IL-4 [20]. In our case, CDA stimulated the release of IL-10 and IFN-γ, suggesting a beneficial immune response that could lead to tolerance. On the contrary, IL-4 was not detected after either treatment, nor with positive control.

The clinical efficacy of immunotherapy has been also associated with the ability of the immunotherapeutic agent to induce IgG antibodies that block IgE-binding sites to the allergens (humoral response) [21]. The results obtained with CDA were positive. CDA extract administered in rabbits produced specific antibodies with capacity to block the IgE binding sites of NE epitopes. This is consistent with the detection of relevant allergens in CDA by mass spectrometry [22]. This means that CDA is captured and processed by the cells of the immune system, producing a specific response (specific IgG) against the allergens combined in the polymerized chains. These specific IgG antibodies generated after the administration of CDA are recognizing and block the natural allergens to which patients are exposed when they are in contact with the allergenic source, in this case, cats. In consequence, the treatment with CDA induces sIgG that blocks IgE binding to the allergen in patients serum [14].

On the other hand, adverse reactions and anaphylaxis as a consequence of IgE reactions remain as the major threat of immunotherapy. Several approaches have been used to reduce immunotherapy-induced side effects. These include immunotherapy with hypoallergens, T-cell epitope-containing peptides, and formulations consisting of Fel d 1 coupled to an immunomodulatory protein or carrier [23]. The polymerization process is the most common method for the reduction of allergenicity because it reduces IgE reactivity, thus improving treatment safety [11]. In our case, the CDA showed a different chromatographic profile compared to NE, characterized by proteins with higher molecular weight. Polymerization also induced a loss of IgE binding, as observed on immunoblot assays, and loss of potency of the CDA extract compared to its corresponding NE, used as a marker of safety. In recent years, new methods based on measurement of the kinetic reaction between antigen and antibody have been postulated as a reliable alternative for the determination of the safety prior to in vivo assays [15]. CDA extract showed more than 4 times less affinity to IgE than NE confirming that the polymerized extract is safer for immunotherapy than native extracts using the same amount of material.

## Conclusions

In summary, we have produced a depigmented and polymerized allergen extract of cat dander to be used as an alternative for cat allergy treatment. The new extract has been designed based on immunological efficacy and safety parameters. The results obtained demonstrate that CDA induces the production of cytokines involved in a Th1 and Treg response (induction of tolerance), is able to induce the production of IgG-blocking antibodies (humoral response), and exhibits reduced interaction with IgE, confirming in vitro efficacy and higher safety than NE. Further in vivo studies should be performed on allergic patients to prove the safety and efficacy of this extract, but the present results suggest that it is a good candidate for the treatment of allergy to cats. New in vitro methods and biomarkers should be optimized in order to design better products for treatments. This work shows the steps followed to evaluate efficacy and safety in vitro during the development of a cat allergoid, which would decrease risks in future clinical trials.

## Abbreviations

CDA: Cat depigmented-polymerized extract
NE: Native Extract
PBMC: Peripheral-blood mononuclear cell
SPR: Surface plasmon resonance
